# Factors influencing birth preparedness and complication readiness during pregnancy in Turkana County, Kenya

**DOI:** 10.11604/pamj.2025.51.82.47999

**Published:** 2025-07-29

**Authors:** Leila Chepkemboi Kibet, Anselimo Ouma Makokha, Joseph Kiplangat Mutai

**Affiliations:** 1 Department of Public Health, Jomo Kenyatta University of Agriculture and Technology, Nairobi, Kenya,; 2 Department of Food Science, Jomo Kenyatta University of Agriculture and Technology, Nairobi, Kenya,; 3 Centre of Public Health Research, Kenya Medical Research Institute, Nairobi, Kenya

**Keywords:** Pregnancy, birth preparedness, obstetric danger signs

## Abstract

**Introduction:**

pregnancy, childbirth, and the postnatal period involve risks that can lead to serious complications, but these can be reduced when mothers and families are aware of key obstetric danger signs and follow a birth preparedness plan.

**Methods:**

a convergent mixed-methods community-based cross-sectional study was conducted among 331 pregnant women in August 2024. Additionally, six key informant interviews and six focus group discussions were held with health workers, pregnant women, and their partners. Quantitative data were analyzed using SPSS v27.0, while qualitative data underwent content analysis. Bivariate and multivariate logistic regressions were employed to identify factors influencing birth preparedness.

**Results:**

only 17.2% of pregnant women were prepared for birth and complications. Awareness of key danger signs was low: 13.5% during pregnancy, 8.9% during labor and childbirth, and 21.9% in the postpartum period. Factors significantly associated with higher odds of birth preparedness included: being a housewife (AOR= 5.6), receiving health education on danger signs (AOR= 4.68), having good knowledge of danger signs during pregnancy (AOR= 3.39), and receiving any form of community support (AOR= 2.74).

**Conclusion:**

birth preparedness and awareness of obstetric danger signs are low among pregnant women in Turkana County. Influencing factors include socio-demographic, cultural, economic, and infrastructural challenges. Qualitative research identified cultural beliefs, the utilization of conventional remedies, and inadequate infrastructure and distance as significant roadblocks to health services. To enhance birth preparedness and complication readiness (BPCR) initiatives in Turkana County, it is recommended that targeted, context-specific health education be implemented during antenatal care clinics. This should involve delivering accurate and relevant health education on obstetric danger signs and the importance of birth preparedness through these clinics. Addressing knowledge gaps among pregnant women and their partners is essential to reduce delays in seeking emergency obstetric care. The Ministry of Health and county governments should expand health facility capacity and provide regular training on focused antenatal care and communication. Community health workers should be recruited and supported to promote antenatal care (ANC) attendance and institutional deliveries through local outreach. To improve access to health centres, county governments and international non-governmental organizations (INGOs) should tackle physical, financial, and social barriers by improving transportation and reaching remote areas.

## Introduction

Complications associated with pregnancy and childbirth remain a substantial public health issue in many countries. Annually, around 300,000 women die owing to complications related to pregnancy or delivery [1]. In 2023, the global maternal mortality ratio was 197 maternal deaths per 100,000 live births, far above the SDGs´ target of fewer than 70 maternal deaths per 100,000 live births by 2030 (WHO *et al*. 2025). In 2023, over 87 percent, equating to 225,000 maternal fatalities, transpired in Southern Asia and sub-Saharan Africa. Southern Asia represented approximately 17 percent (43,000 fatalities), but sub-Saharan Africa constituted nearly 70 percent (182,000 fatalities). The World Health Organization indicates that elevated maternal death rates signify substantial disparities in access to quality healthcare services. Women frequently succumb to difficulties that occur during and after pregnancy and childbirth. Many of these issues arise during pregnancy and can be managed or averted by suitable medical intervention [1].

The maternal mortality ratio in Kenya is high at 530 deaths per 100,000 live births (World Bank, 2024), despite the Kenyan government's recent attempts to enhance obstetric outcomes. Since 2020, 7764 Kenyan women have died during childbirth, according to the UNICEF report 'The Future of Childhood in a Changing World Statistical Compendium,' which was released in 2025. This pattern, which is particularly evident in low-income nations like Kenya, suggests that both access to and quality of antenatal, delivery, and postpartum care will need to be improved [2]. To curb maternal morbidity and death rates, governments and international organizations are developing and executing many policies, programs, and interventions. One strategy is birth preparedness and complication readiness, which is becoming recognized in developing countries as a means to mitigate obstetric risks for both mothers and infants. This method encourages the utilization of competent delivery personnel and appropriate medical facilities, along with health-seeking behavior. The main idea is that careful planning and preparation for obstetric complications can help pregnant women and their families access timely healthcare when needed [3,4]. Initially, it advocates for early birthing planning by promoting the engagement of a qualified healthcare professional during delivery. This proactive strategy mitigates the first delays in the decision to pursue care and in accessing a healthcare facility. These delays are often the result of financial constraints, limited access to healthcare services, and inadequate awareness among families and communities regarding maternal and neonatal health issues. When women and their families opt to pursue medical care before the commencement of labor and effectively execute that decision, it facilitates prompt care before difficulties arise. Further delays may result from inadequate road conditions, considerable distances to healthcare facilities, and the lack of emergency transportation.

Birth preparedness and complication readiness enhance the identification of health issues and promote the timely decision to seek medical attention by increasing awareness of warning indicators among pregnant women, their families, and their communities. It encourages families and communities to take practical measures, including identifying individuals who could donate blood in the event of an emergency, preserving money for medical and travel expenses, and arranging transportation. This level of preparedness enables the rapid response to complications and guarantees that critical decisions are made without unnecessary delays. The beneficial effects of BPCR interventions on maternal and neonatal health are corroborated by research evidence. According to [5], exposure to such interventions resulted in an 18 percent decrease in neonatal fatalities and a 28 percent decrease in maternal deaths.

The Ministry of Health in Kenya has implemented the WHO's focused antenatal care guidelines to ensure the health and survival of mothers and their infants. These guidelines promote the implementation of structured antenatal visits that prioritize the identification, prevention, and management of severe health conditions during pregnancy, as well as the preparation of women and their families for birth and potential emergencies. These visits now function as an entry point for a wide variety of integrated health services [6]. In Kenya, the FANC includes BPCR packages. Consequently, during birth preparedness counseling at ANC, women are encouraged to select a secure and reputable delivery location, appoint a skilled birth attendant, identify potential blood donors in the event of hemorrhage, save emergency funds, be aware of the anticipated delivery date, screen for HIV/AIDS, purchase essential birthing supplies, and arrange for emergency transportation.

Turkana County's Integrated Development Plan for 2018-2022 [7] indicates that the maternal mortality rate is greater than 1000 per 100,000 live births [7]. Other health indicators in Turkana are below the national average, such as the proportion of skilled deliveries, which is currently 43%. There is a dearth of research on maternal health in Turkana, and none has investigated the factors that affect the readiness of pregnant women for childbirth and complications in Turkana County. The objective of this investigation is to assess the factors that influence the readiness of expectant women to deal with complications and birth preparation in Turkana East Sub-County, which is one of the sub-counties of Turkana County. The results from this study will be highly beneficial for health planners and decision-makers, including the Ministry of Health, county government, and national and international agencies, in guiding appropriate interventions and developing useful programs to raise pregnant women's preparedness for complications and birth. The World Health Organization also promotes BPCR as a key strategy to increase skilled care utilization and timely management of obstetric and newborn complications [8]. However, there remains a lack of published evidence on BPCR prevalence and associated factors in Turkana, which this study seeks to address.

## Methods

**Study design and setting:** this study employed a mixed-method convergent parallel design, integrating both quantitative and qualitative approaches. Data collection methods included structured questionnaires with closed- and open-ended questions, in-depth interviews guided by a protocol, and structured observations using checklists. The research was conducted in Turkana County, northwestern Kenya, the largest of Kenya´s 47 counties by land area, primarily inhabited by nomadic pastoralists. Turkana is characterized by an arid and semi-arid climate with widespread challenges such as limited healthcare access, high maternal mortality, malnutrition, insecurity due to cattle rustling, poverty, famine, and cultural practices that complicate maternal and child health outcomes. The county is divided into six sub-counties: Kibish, Loima, Turkana Central, Turkana East, Turkana South, and Turkana North, with a total population of approximately 926,976 [9]. Health interventions by the government and NGOs, including mobile clinics and community health programmes, aim to improve health access despite logistical difficulties.

**Study population and sampling:** the study targeted pregnant women residing in selected Turkana villages for at least six months, regardless of pregnancy trimester. Additionally, partners of pregnant women and healthcare providers were involved through Focus Group Discussions (FGDs) and Key Informant Interviews (KIIs). Exclusion criteria included critical illness, inability to respond, or refusal to consent. For the quantitative component of the study, Turkana East Sub-County was randomly selected from among the six sub-counties. A multistage sampling strategy was employed to identify participants. First, the three wards within the sub-county, namely Lokori, Katilia, and Napeitom, were purposively selected and treated as strata, each allocated an equal sample size. Within these wards, a total of six villages were selected, and systematic sampling was used to identify pregnant women for interviews. The number of participants per village was determined proportionally based on population size. Household lists were developed with support from local elders and community health volunteers. The first household in each village was selected through simple random sampling, after which subsequent households were identified systematically until the target sample size was reached. In cases where multiple eligible pregnant women were found in a single household, one was randomly selected to participate. Following the interviews, participants were invited to join focus group discussions (FGDs), held in commonly used community spaces such as shaded areas near seasonal riverbeds. Male partners participated in separate FGDs conducted at local administrative offices. The qualitative sample comprised six FGDs; four with pregnant women and two with their male partners, as well as six key informant interviews (KIIs) with healthcare workers, who were purposively selected based on their roles in maternal health service delivery. Due to the limited number of healthcare personnel in the selected sub-county, only six were available and consented to participate. All FGDs and KIIs were scheduled one week in advance, and informed consent was obtained from all participants before data collection.

**Sample size determination:** the quantitative sample size was calculated using the single population proportion formula by Fischer (1991) [10]:


$$n=\frac{{\left(Z\alpha /2\right)}^{2}*p*\left(1-p\right)}{d^{2}}$$

Where n= the desired sample size; Z_α/2_= standard Normal deviate usually set at 1.96, corresponding to 95%, p=0.28 (proportion of birth preparedness estimated from a previous East Pokot study [11], and d=0.05 (margin of error). This yielded a required sample size of 310, adjusted to 331 to account for a 10% non-response rate.

**Data collection tools and procedures:** quantitative data were collected using a pretested questionnaire administered via mobile phones using the KOBO Collect application. The questionnaire addressed socio-demographic, economic, obstetric, socio-cultural, and healthcare-related factors influencing birth preparedness and complication readiness. It was pretested on 35 pregnant women in Lokori town, within the study area, to ensure clarity and improve validity; ambiguous questions were revised accordingly. Questions were also reordered for logical flow. Data clerks were trained and conducted pretesting to enhance data quality. Qualitative data collection employed semi-structured focus group discussions and KII guides focusing on themes such as BPCR, antenatal care utilization, male involvement, traditional birth attendance, barriers to care, and recommendations from mothers. Focus group discussions were conducted in community meeting areas, facilitated by the principal investigator and a bilingual assistant. KIIs with healthcare workers took place at health facilities during convenient times, such as lunch breaks. All qualitative sessions were audio-recorded, transcribed verbatim, and translated by bilingual experts to preserve accuracy.

**Study variables:** the definition of key variables used in this study is provided in Table 1.

**Table 1:** definition of key variables used in the study

Variables	Description
Birth preparedness and complication readiness prepared	The following essential components were used to evaluate birth preparedness and complication readiness (BPCR) actions: choosing a preferred delivery location (and a trained birth attendant); setting aside funds for birth-related expenses; arranging transportation to a medical facility in case of emergencies; finding a compatible blood donor; being aware of obstetric danger signs; locating a medical facility that offers 24-hour emergency obstetric care in case of obstetric emergencies, and preparing necessary supplies, such as clean clothes and other materials; setting up an emergency fund; developing a communication plan in case it becomes necessary to contact ambulances or other support; determining the expected date of delivery; and choosing a support person to accompany the expectant mother to the medical facility for delivery. A pregnant woman was deemed prepared if she met at least six of these ten criteria BPCR requirements.
Awareness of obstetric danger signs	When a woman mentioned at least three of the major pregnancy danger signs: vaginal bleeding, swollen hands or face, blurred vision, and a loss of liquor, she was deemed knowledgeable about these signs. When a woman indicated at least three important labor and delivery danger signals (severe vaginal bleeding, protracted labor >12 hours), the placenta was not delivered 30 minutes after the baby was born, and convulsions occurred; she was deemed informed about these signs. When a woman cited at least three of the main postpartum danger signs, severe vaginal bleeding, foul-smelling vaginal discharge, and a high fever, she was deemed knowledgeable about these symptoms.

**Data analysis:** quantitative data were reviewed daily for completeness, coded, and analyzed using IBM SPSS version 27. Descriptive statistics summarized participants´ socio-demographic and obstetric characteristics. Bivariate analyses, including Chi-square, identified associations between BPCR and independent variables. Variables with p-values <0.25 and clinically relevant factors were included in multivariable binary logistic regression models using stepwise selection. Adjusted odds ratios with 95% confidence intervals were reported, and statistical significance was set at p <0.05. Qualitative data were analyzed concurrently using NVivo 11 software. Content analysis was applied with both deductive and inductive coding. The principal investigator performed coding, grouping codes into subcategories and main themes. Participant quotes were preserved verbatim, including vernacular expressions, and pseudonyms were used to maintain confidentiality.

**Ethical considerations:** ethical approval was obtained from the Kenya Medical Research Institute´s Scientific Ethical Review Unit (KEMRI SERU), Jomo Kenyatta University of Agriculture and Technology Graduate School, the National Commission for Science, Technology and Innovation (NACOSTI), and relevant county health authorities. Informed consent was obtained from all participants following a full briefing on the study´s purpose, procedures, and associated risks and benefits.

## Results

### Socio-demographic characteristics of the respondents

As shown in Table 2, A total of 331 pregnant women participated in the study, aged 15-39 years (median 25). Most were married (87%) and Christian (85.8%). Nearly half (45.9%) had no formal education; others completed primary (19.9%), secondary (23.3%), diploma (10.6%), or university (0.3%). Over half (54%) were housewives without income, and nearly half of married women´s husbands were pastoralists. About 40% had relocated recently due to nomadic pastoralism or drought. Forty-two percent were pregnant for the first time, while 58% were multiparous. Most were in their second (50.5%) or third trimester (44.4%). Previous stillbirths were reported by 16.5%. Delivery plans included health centers (59.6%), referral hospitals (28.9%), and home births (11.5%). Antenatal care attendance was high (86.4%), with non-attendance mainly due to family disapproval, distance, and care dissatisfaction. Travel to facilities typically took 30-60 minutes, mostly by motorbike, walking, or public transport. Some women did not plan facility deliveries.

**Table 2:** sociodemographic characteristics of pregnant women (N = 331) in Turkana County

Variable	Number	Percent
**Age**		**Median 25.0; range 24(15-39) IQR 9(20-29)**
**Age categories**		
15-24	147	44.4
25-34	168	50.8
≥35	16	4.8
**Marital status of the respondent**		
Divorced	2	0.6
Married	288	87.0
Separated	22	6.6
Single	17	5.1
Widowed	2	0.6
**Religion of the respondent (n=330)**		
Christianity	283	85.8
Islam	16	4.8
Traditional African religion	31	9.4
**Highest level of education of the respondent**		
Degree and above	1	0.3
Diploma, Certificate, TVET (same category)	35	10.6
No formal education	152	45.9
Primary education	66	19.9
Secondary education	77	23.3
**Occupation of the respondent**		
Business	119	36.0
Employed	29	8.8
Farmer	1	0.3
Housewife	178	53.8
Pastoralist	4	1.2
**Occupation of the respondent’s husband (skip if no husband) (n=293)**		
Agro pastoralist	2	0.7
Business	67	22.9
Employed	69	23.5
Farmer	1	0.3
Not working	14	4.8
Pastoralist	140	47.8
**Family movement in the last 2 years (n=330)**		
Yes	131	39.7
No	199	60.3
Reasons for family movement (n=131)		
Nomadic pastoralism (n=131)	91	69.5
Natural disasters such as drought conditions (n=131)	60	45.8
Other reasons (n=131)	16	12.2
**Household income of the respondent per month (Kshs) (n=330)**		
4000 and below	132	40.0
5000 to 15000	113	34.2
Above 16,000	85	25.8

IQR: interquartile range; Kshs: Kenya shillings

Qualitative data from key informant interviews and focus group discussions highlighted significant barriers to accessing health services, including long distances, poor transport infrastructure, financial constraints, and insecurity. These challenges affect women´s ability to consistently access ANC and delivery services, impacting maternal health outcomes. Some of the participants had the following to say:…"*It takes us approximately 3 hours to reach the dispensary by foot or pay shillings five hundred (KES 500) to a motorcycle operator to reach there within 30 minutes*"(FGD,2). “*... these areas have been reporting cases of insecurity which hinders women from going to seek assistance from health centres*.” (KII,4).

### Awareness of obstetric key danger signs associated with birth preparedness and complication readiness practices among pregnant women

As indicated in Table 3, in the survey, 78.5% of participants understood that unforeseen problems during pregnancy or childbirth could threaten a woman´s life. Furthermore, 86.4% reported being aware of specific danger signs related to pregnancy and childbirth. Among those aware, knowledge was gained from various sources: 17.8% from personal experience, 53.1% from Community Health Workers or Volunteers, 67.8% from healthcare workers and health facilities, 63.3% from other women, 43.7% from NGOs, 33.2% from family members, and 20.6% from mass media like radio or television. In-depth interviews with healthcare providers revealed that birth preparedness information is generally shared during antenatal care visits. However, some women miss this information due to irregular ANC attendance or inconsistent communication by health workers. Additionally, not all pregnant women receive education on obstetric danger signs when visiting health facilities, limiting their awareness. “*It depends on the health officer who is providing the ANC services, sometimes it is discussed, and sometimes it is not*,” (FGD, 5). “*Some women do not go for ANC services and miss out on crucial information about obstetric danger signs*,” (FGD,1). The study also established that certain traditional practices, such as using herbal remedies and rituals, influence maternal health behaviours. These practices can delay seeking professional healthcare and complicate birth outcomes. “*There are traditional practices like using specific herbs and dietary restrictions. Mothers remain indoors for at least 10 days after childbirth*,” (KII,6). Cultural beliefs sometimes conflict with modern medical advice, underscoring the need for culturally sensitive interventions that address these practices while promoting safe birth practices.

**Table 3:** proportion of pregnant women who reported types of obstetric danger signs during pregnancy, childbirth, and postpartum period

Obstetric danger signs	Pregnancy (n=327)	Labor and childbirth (n=327)	Two days after childbirth (postpartum) (n=329)
	**n(%)**	**n(%)**	**n(%)**
Vaginal bleeding	308(94.2)	243(74.3)	293(89.1)
Convulsions/loss of consciousness	128(39.1)	130(39.8)	162(49.2)
Severe headache	111(33.9)	60(18.3)	-
Severe abdominal pain	146(44.6)	-	171(52)
High fever	81(24.8)	-	137(41.6)
Swollen hands and face	113(34.6)	-	-
Blurred vision	100(30.6)	-	-
Lack of fetal movement	234(71.6)	-	-
Difficulty breathing	115(35.2)	-	-
Water breaks before labor	177(54.1)	-	-
Prolonged labor (more than 12 hours)	-	261(79.8)	-
Retained placenta	-	202(61.8)	-
The hands/feet of the fetus appear first	-	209(63.9)	-
Severe vaginal bleeding	-	-	293(89.1)
Foul-smelling vaginal discharge	-	-	198(60.2)
Swollen hands and face	-	-	87(26.4)
Other	-	-	4(1.2)
Knowledgeable of key danger signs	44(13.5)	29(8.9)	72(21.9)
Less knowledge of key danger signs	283(86.5)	298(91.1)	257(78.1)

### Knowledge of obstetric danger signs across the 3 phases among pregnant women in Turkana County

As shown in Table 3, participants identified vaginal bleeding as the most critical danger sign during pregnancy, mentioned by 94.2%. Other key signs included lack of fetal movement (71.6%), water breaking before labor (54.1%), severe abdominal pain (44.6%), swollen hands and face (34.6%), convulsions (39.1%), and blurred vision (30.6%). During labor and childbirth, prolonged labor (over 12 hours) was the most frequently reported danger sign (79.8%), followed by vaginal bleeding (74.3%), head or feet of the fetus appearing first (63.9%), retained placenta (61.8%), and convulsions (39.8%). After birth, severe vaginal bleeding (89.1%) was again the most commonly cited problem, along with foul-smelling vaginal discharge (60.2%), severe abdominal pain (52%), and high fever (41.6%). Women were considered knowledgeable if they mentioned at least five danger signs across the three phases: pregnancy, labor/childbirth, and postpartum, with at least one danger sign from each phase. Those mentioning fewer signs had low or no knowledge. The study found that most women had low knowledge of danger signs: 86.5% during pregnancy, 91.1% during labor and childbirth, and 78.1% during postpartum.

**Level of birth preparedness and complication readiness practices among pregnant women in Turkana County:** this study assessed birth preparedness among pregnant women using the WHO´s eleven key components of birth preparedness namely; identified skilled attendants to assist at birth, identified a health facility for emergency, identified blood donor in case of emergency, made arrangement for transport to the health facility, saved money in case of any expenses, identified health institution with 24hr emergency obstetric care, prepared clean clothes and other materials, bought and prepared materials for childbirth including food, knowledge of expected date of delivery and identified who would accompany them during emergency, prepared clothes and other materials, identified obstetric danger signs and identified a place for giving birth. Women who completed six or more components were considered well-prepared, but only 17.2% met this threshold, while the majority (82.8%) were less prepared. Most women (89%) also showed limited knowledge of birth preparedness and its importance. Despite this, many women took some steps to prepare for delivery: around 74% identified a place for delivery and prepared clean clothes, 58% saved money, and 57% arranged transport for emergencies. However, fewer women had identified support people (33%), blood donors (4%), or a 24-hour emergency health facility (3%), indicating significant gaps in comprehensive birth preparedness among the participants (Table 4).

**Table 4:** birth preparedness and complication readiness among pregnant women (n=331) in Turkana County

Levels of birth preparedness and complication readiness actions/steps taken	Yes(n,%)	No(n,%)
Identified place for giving birth	246(74.3%)	85(25.7%)
Saved money in case of any expenses	192(58%)	139(42%)
Identified means of transport to the health facility in case of an emergency	189(57.1%)	142(42.9%)
Identified blood donor in case blood will be needed when I am giving or after birth	13(3.9%)	318(96.1%)
Identified emergency obstetric signs	4(1.2%)	327(98.8%)
Identified a health institution with 24-hour emergency obstetric care	10(3%)	321(97%)
Prepared clean clothes and other materials	247(74.6%)	84(25.4%)
Arranged for an emergency fund	15(4.5%)	316(95.5%)
Bought and prepared materials for childbirth, including food	157(47.4%)	174(52.6%)
Identified support people to help, such as friends and family	109(32.9%)	222(67.1%)
I know my expected date of delivery	80(24.2%)	251(75.8%)
Number of steps taken	n	%
0	6	1.81
1	16	4.83
2	50	15.11
3	75	22.66
4	77	23.26
5	50	15.11
6	44	13.29
7	9	2.72
8	2	0.60
9	0	0.00
10	2	0.60
11	0	0.00
Less-prepared (<6 arrangements made)	274	82.78
Well prepared (≥ 6 arrangements made)	57	17.22

### Enablers and barriers associated with birth preparedness practices among pregnant women

**Barriers**

**Limited knowledge and practice of birth preparedness:** as indicated in Annex 1, both women and men showed poor knowledge and inconsistent birth preparedness practices. Many believed attending antenatal care (ANC) was sufficient preparation, without recognizing other important steps recommended by WHO. For example, some women mentioned saving food, setting aside funds, or preparing a hospital bag, but awareness of comprehensive birth planning was limited: “*For us women, we attend all ANC visits and carry our hospital bags full of baby clothes and other things we need after giving birth*,” (FGD,3). “*For us men, preparation means providing good, nutritious food*,” (FGD,7). Information about birth preparedness during ANC visits was inconsistent. Some women reported receiving education, while others said the topic was rarely or unevenly covered: “*Sometimes birth preparedness is discussed, sometimes not. It depends on the health worker that is on duty during clinic days*,” (FGD,6). Most pregnant women believed birth preparation should begin between the 6th and 8th month, indicating a lack of early awareness.

**Partners' knowledge gaps:** most male partners had limited knowledge of danger signs because they do not attend ANC clinics. One man admitted: “*We do not know danger signs unless our wives tell us*,” (FGD,8).

**Distance and access challenges:** the study took place in the remote, insecure Turkana East Sub-County, where distance and poor infrastructure severely limit access to ANC and skilled birth attendance. Over half the women walked up to an hour to reach a facility; 51.1% cited distance as the main reason for missed ANC visits. A healthcare worker noted: “*Many pregnant women live miles from facilities and cannot reach hospitals when labor starts*,” (KII,4). FGDs cited insecurity, poverty, and lack of transport as additional barriers: “*A motorbike ride costs 500 shillings, which we cannot afford, so we walk despite security challenges on the road and the long distances to the health centers*,” (FGD,3). Women often resorted to home births or traditional practitioners due to transport or financial constraints.

**Health facility resource shortages:** healthcare workers and women reported frequent shortages of essential supplies (antibiotics, cotton wool, and oxytocin), hampering effective management of complications. A healthcare worker said: “*Managing complications is difficult due to a lack of delivery supplies*” (KII,1). Pregnant women in FGD highlighted the lack of ambulances for emergency referral: “*There are few or no ambulances; the referral hospital is over a day away, and terrain and cost make transport very difficult. Many prefer herbs for complications*” (FGD,1).

**Cultural influences:** cultural beliefs restrict maternal health care-seeking, including postpartum confinement and dietary restrictions. Pastoral mobility further reduces ANC attendance, and many women are restricted by husbands or in-laws from attending clinics. These factors hinder timely care and health education.

### Factors associated with birth preparedness and complication readiness among the respondents

The socio-demographic characteristics influencing birth preparedness and complication readiness practices among pregnant women in Turkana County were analyzed. Bivariate results showed that women affiliated with the Islamic religion (OR= 2.94, 95% CI= 1.02-8.47; p= 0.046) and those who were employed (OR= 4.98, 95% CI= 1.94-12.83; p= 0.001) were more likely to be well prepared. However, multivariable analysis adjusting for various factors revealed that being a housewife was significantly associated with better BPCR practices (aOR= 5.60; 95% CI= 2.21-14.19; p<0.001) (Table 5).

**Table 5:** sociodemographic factors associated with the practice of birth preparedness and complication readiness among pregnant women in Turkana County

Variable	Birth preparedness and complication readiness practice					
	Yes (well prepared)	No (less-prepared)	OR (95%CI)	P-value	AOR (95%CI)	P-value
**Age category**						
15-24	29(19.7%)	118(80.3%)	1.0	0.368	1.0	0.361
25-34	27(16.1%)	141(83.9%)	0.78(0.44-1.39)	0.398	1.13(0.51-2.47)	0.769
≥35	1(6.3%)	15(93.8%)	0.27(0.03-2.14)	0.216	0.19(0.02-2.11)	0.176
**Religion of the respondent**						
Christianity	48(17.0%)	235(83.0%)	1.0	0.068		
Islam	6(37.5%)	10(62.5%)	2.94(1.02-8.47)	0.046		
Traditional African religion	3(9.7%)	28(90.3%)	0.52(0.15-1.80)	0.304		
**Occupation of the respondent**						
Business	13(10.9%)	106(89.1%)	1.0	0.024	1.0	0.008
Employed	11(37.9%)	18(62.1%)	4.98(1.94-12.83)	0.001	2.11(0.55-8.07)	0.275
Farmer	0(0.0%)	1(100.0%)	ND	1.000	ND	1.000
Housewife	32(18.0%)	146(82.0%)	1.79(0.90-3.57)	0.100	5.60(2.21-14.19)	<0.001
Pastoralist	1(25.0%)	3(75.0%)	2.72(0.26-28.08)	0.401	14.93(0.64-348.07)	0.092

OR: odds ratio; AOR: adjusted odds ratio; CI: confidence interval; ND: no data

As indicated in Table 6 and Table 7, obstetric characteristics also played a crucial role in BPCR. Bivariate analysis indicated that Muslim women, those in all three trimesters, those attending antenatal care (ANC) and receiving blood pressure monitoring service, those receiving health education on danger signs, those given health education on general health and nutrition, and those planning to use private or public transport for delivery showed higher preparedness. After adjustment, attending ANC receiving health education on pregnancy danger signs (aOR= 4.68; 95% CI= 1.52-14.45; p= 0.007), and planning to use private transport for delivery (aOR= 13.01; 95% CI= 1.97-86.11; p= 0.008) remained significantly associated with BPCR. Knowledge of obstetric danger signs was strongly linked to BPCR practices. Bivariate analysis showed that women aware of potential pregnancy complications and key danger signs during pregnancy and in newborns were more prepared. Multivariable results further confirmed that knowledge of danger signs during pregnancy (aOR=3.39; 95% CI=1.10-10.38; p=0.033), receiving health education on danger signs (aOR=30.12; 95% CI=1.33-683.45; p=0.033), education on birth preparedness (aOR=12.09; 95% CI=1.34-109.35; p=0.027), and community support in birth preparation (aOR=2.74; 95% CI=1.14-6.58; p=0.024) were significant predictors of BPCR practices (Table 6 and Table 7).

**Table 6:** obstetric factors associated with the practice of birth preparedness and complication readiness among pregnant women in Turkana County

		Birth preparedness and complication readiness practice					
Variable		Yes (well prepared)	No (less-prepared)	OR (95%CI)	P-value	AOR (95%CI)	P-value
Trimester at the time of the interview	First	7(41.2%)	10(58.8%)	1.0	0.040	1.0	0.341
	Second	27(16.2%)	140(83.8%)	0.28(0.10-0.79)	0.016	0.35(0.08-1.52)	0.160
	Third	23(15.6%)	124(84.4%)	0.26(0.09-0.77)	0.014	0.48(0.11-2.08)	0.328
Is this your first pregnancy?	No	33(17.2%)	159(82.8%)	1.0		1.0	
	Yes	24(17.3%)	115(82.7%)	1.01(0.56-1.79)	0.985	1.48(0.64-3.40)	0.360
Attended antenatal care (ANC) and given blood pressure monitoring	No	1(2.0%)	50(98.0%)	1.0			
	Yes	55(23.4%)	180(76.6%)	15.28(2.06-113.16)	0.008		
Attended ANC and given Health education on danger signs of pregnancy	No	8(7.5%)	98(92.5%)	1.0		1.0	
	Yes	48(26.7%)	132(73.3%)	4.45(2.02-9.84)	<0.001	4.68(1.52-14.45)	0.007
Attended ANC and given Health education on general health and nutrition	No	19(12.2%)	137(87.8%)	1.0		1.0	
	Yes	37(28.5%)	93(71.5%)	2.87(1.55-5.29)	0.001	1.48(0.64-3.42)	0.356
Means of transport respondents plan to use to get to the health centre/hospital to deliver the current pregnancy	Walking on foot	4(6.0%)	63(94.0%)	1.0	<0.001	1.0	0.027
	Ambulance	0(0.0%)	1(100.0%)	ND	1.000	0.00	1.000
	I don’t plan to go to the hospital/health centre	0(0.0%)	21(100.0%)	ND	0.998	ND	ND
	Motorbike	21(14.9%)	120(85.1%)	2.76(0.91-8.38)	0.074	1.85(0.41-8.34)	0.422
	Private means	14(51.9%)	13(48.1%)	16.96(4.80-59.88)	<0.001	13.01(1.97-86.11)	0.008
	Public means	18(28.6%)	45(71.4%)	6.30(2.00-19.88)	0.002	3.71(0.77-17.79)	0.102

OR: odds ratio; AOR: adjusted odds ratio; CI: confidence interval; ND: no data

**Table 7:** awareness of obstetric key danger signs associated with BPCR practice

	BPCR practice					
Variable	Yes (well prepared)	No (less-prepared)	OR (95%CI)	P-value	AOR (95%CI)	P-value
Unforeseen problems related to pregnancy can occur during any pregnancy or childbirth situation that could endanger the life of a woman						
No	6(8.5%)	65(91.5%)	1.0			
Yes	51(19.6%)	209(80.4%)	2.64(1.09-6.44)	0.032		
Know the danger signs of pregnancy and childbirth						
No	4(8.9%)	41(91.1%)	1.0			
Yes	53(18.6%)	232(81.4%)	2.34(0.80-6.82)	0.119		
knowledge of key danger signs during pregnancy						
Less knowledge of key danger signs	43(15.2%)	240(84.8%)	1.0		1.0	
Knowledgeable of key danger signs	14(31.8%)	30(68.2%)	2.60(1.28-5.31)	0.008	3.39(1.10-10.38)	0.033
knowledge of key danger signs during childbirth						
Less knowledge of key danger signs	48(16.1%)	250(83.9%)	1.0		1.0	
Knowledgeable of key danger signs	9(31.0%)	20(69.0%)	2.34(1.01-5.46)	0.048	1.60(0.41-6.32)	0.499
Knowledge of key danger signs in postpartum						
Less knowledge of key danger signs	46(17.9%)	211(82.1%)	1.0		1.0	
Knowledgeable of key danger signs	11(15.3%)	61(84.7%)	0.83(0.40-1.69)	0.604	0.58(0.22-1.50)	0.261
Knowledgeable of key danger signs	3(75.0%)	1(25.0%)	14.94(1.53-146.39)	0.020	1.10(0.07-18.47)	0.946
Received health education on danger signs of pregnancy, childbirth, and after birth						
No	1(2.2%)	45(97.8%)	0.090(0.01-0.67)	0.019	30.12(1.33-683.45)	0.033
Yes	56(19.7%)	228(80.3%)	1.0		1.0	
Received health education on how to prepare for birth and its complications						
No	2(1.6%)	122(98.4%)	1.0		1.000	
Yes	55(26.6%)	152(73.4%)	22.07(5.28-92.31)	<0.001	12.09(1.34-109.35)	0.027
Community assists pregnant women in whichever way to prepare for birth						
No	34(16.6%)	171(83.4%)	0.89(0.50-1.59)	0.696	2.74(1.14-6.58)	0.024
Yes	23(18.3%)	103(81.7%)	1.0		1.0	

OR: odds ratio; AOR: adjusted odds ratio; CI: confidence interval; BPCR: birth preparedness and complication readiness

## Discussion

### Awareness of birth preparedness and complication readiness

This study found that only 11% of pregnant women demonstrated adequate knowledge of birth preparedness and complication readiness (BPCR), aligning with [12] in Somaliland but considerably lower than reports from Enugu, Nigeria (59%) and South Western Ethiopia (97.1%) [13,14]. The disparity likely reflects the rural setting of this study and Somaliland, where access to health information is limited compared to urban populations with greater exposure to healthcare providers and media. Key BPCR components: identifying a place for childbirth (81.8%), preparing clean materials (75.6%), arranging transportation (66.7%), and saving money (61.2%) were widely recognized. However, awareness of emergency readiness aspects, such as blood donor identification (5.8%) and recognizing obstetric danger signs (6.5%), was markedly low. These findings align with [12], although their participants showed higher recognition of blood donor identification.

Regional comparisons indicate that knowledge in this study surpasses that of semi-pastoral pregnant women in Southern Ethiopia [15], possibly reflecting Kenya´s Focused Antenatal Care (FANC) programs that include group education on BPCR during antenatal visits. However, awareness of delivery place identification remains lower than in the Bench Maji Zone, South West Ethiopia, where 85.2% of women acknowledged its importance [14]. These findings highlight the need for context-specific health education and service delivery strategies to enhance maternal preparedness in rural, resource-limited settings.

### Practices of birth preparedness and complication readiness

Only 17.2% (57 women) met the criteria for adequate BPCR practice, with 82.8% classified as ‘less prepared.´ This low prevalence is consistent with regional findings from Cameroon (18.8%) [16], Tanzania and Kenya (11.4% and 7.6%, respectively) [17], Southwest Ethiopia (24.3%) [14], and Rwanda (22.3%) [18]. Higher rates were reported in facility-based studies in Kenya (70.5%) and Tanzania (58.4%) [19,20], likely reflecting greater urban exposure to health information. Among BPCR components, delivery place identification was most common (74.3%), consistent with Tanzania (77.8%) [20], Southwest Ethiopia (83.7%) [14], and Cameroon (80%) [16]. Conversely, lower figures were reported in Rwanda (19.4%) and Ethiopia (10.1%) [18,21]. Preparation of clean clothes (74.6%) and saving money for delivery (58%) were also prevalent, aligning with Somaliland (85% and 67.7%) [12] and Nepal (38.5%) [22]. Transportation arrangements were made by 57.1%, comparable to Cameroon (57.1%) [16] and Nepal (50.7%) [22] but higher than Rwanda [18] and East Pokot, Kenya [23].

Identification of a compatible blood donor was among the least practiced actions (3.9%), despite postpartum hemorrhage being a leading cause of maternal mortality [24]. Similar low rates were noted in Somaliland (10.3%), Kenya (1%), and Tanzania (3%) [12,17]. Despite high awareness of obstetric danger signs, only a small fraction arranged for emergency blood donors, indicating insufficient counselling during antenatal care. Identification of support persons was low (32.9%), and only 24.2% were aware of their expected delivery dates, underscoring gaps in comprehensive preparedness. Overall, these findings reveal critical deficiencies in BPCR practices, especially in rural areas with socioeconomic, cultural, and infrastructural barriers. Such gaps hinder timely decision-making and access to skilled care, essential for reducing maternal morbidity and mortality. Addressing these requires enhanced antenatal counselling, improved health system access, and community-targeted interventions.

### Awareness of key obstetric danger signs

Knowledge of obstetric danger signs was low: 13.5% demonstrated adequate knowledge during pregnancy, 8.9% during labour and childbirth, and 21.9% postpartum. Most knowledgeable women reported information sources as healthcare workers (67.8%), other women (63.3%), community health workers (53.1%), and community meetings or NGOs (43.7%). This low awareness mirrors findings in Somaliland (35.3%, 28%, and 39.5% for the three phases) [12], Southwest Ethiopia (55.9%, 24.6%, and 43.6%) [14], and Kenya (34.2% aware of two or more signs during pregnancy) [19,25]. In Asia, reported awareness was below 23% across all phases. Conversely, a rural hospital study in Rwanda [18] showed higher recognition, with over 75% identifying at least one danger sign in each phase, yet comprehensive knowledge (three or more danger signs throughout pregnancy, labour, and postpartum) was low at 6.6% [18].

Such deficiencies increase risks of delayed care-seeking and poor birth preparedness, linked to maternal morbidity and mortality [26]. Strengthening awareness initiatives for women of reproductive age is crucial. Vaginal bleeding was the most recognized danger sign: 94.2% during pregnancy, 74.3% during childbirth, and 89.1% postpartum, consistent with Rwanda [18] and Somaliland [12]. Lower awareness was reported in Kenya [19]. The high recognition likely reflects its severity. Nonetheless, awareness of one danger sign is insufficient; comprehensive knowledge and prompt responses are vital to safeguarding maternal and newborn health.

### Relationship between sociodemographic, obstetric factors, awareness, and BPCR practices

Occupation significantly influenced BPCR: housewives were 5.6 times more likely to be prepared compared to farmers or employed women (aOR= 5.60; 95% CI= 2.21-14.19; p<0.001). This contrasts with Cameroon, where government-employed women had higher preparedness [16]. The difference may stem from active community health workers and NGOs in Turkana providing maternal health education through household visits and meetings, which housewives attend more frequently. Obstetric factors linked to BPCR included attending antenatal care (ANC) with health education on pregnancy danger signs (aOR= 4.68; 95% CI= 1.52-14.45; p= 0.007). Aligning with studies from Ethiopia [27], Kenya [28], Tanzania [20], and others [17,21,29] emphasized that ANC quality and consistency, not just frequency, drive BPCR improvements, a point supported by Turkana qualitative data showing inconsistent ANC messages. Improving ANC quality and community interventions like health outreaches are essential.

Good knowledge of obstetric danger signs was strongly linked to BPCR; knowledgeable women were 3.4 times more likely to be prepared (aOR=3.39; 95% CI=1.10-10.38; p=0.033). Knowledge was measured by the ability to name at least six danger signs across pregnancy, childbirth, and postpartum phases. Similar findings are reported in Tanzania, Rwanda, Ethiopia, and rural Ethiopia ([14,18,20,21]. Variations in other studies [17,30] may reflect differences in populations and timing of data collection, underscoring the need for targeted education and counselling during ANC visits.

Cultural practices and barriers such as long distances and poor transport also hinder timely healthcare seeking, as reported in this and other studies [31,32]. Community involvement predicted BPCR (aOR= 2.74; 95% CI= 1.14-6.58; p= 0.024), consistent with Nigerian behavioural intervention findings [33]. Engagement of partners, families, community health workers, and stakeholders through advocacy, training, emergency fund and transport schemes, and educational materials improved birth preparedness and danger sign knowledge. This underscores the importance of multistakeholder involvement, including men, families, communities, and healthcare providers in maternal health programs [34].

**Strengths and limitations:** this study fills a significant gap in maternal health equity research by focusing on underserved nomadic populations. Another strength lies in its convergent mixed-methods approach that allows triangulation and contextual richness. Thirdly, the inclusion of male partner perspectives and cultural dimensions adds community-level relevance. A key limitation is reliance on self-reported data for BPCR assessment, which may be affected by social desirability bias. However, qualitative data corroborated quantitative findings, mitigating this concern.

## Conclusion

This study demonstrates that practices of birth preparedness and complication readiness are low among pregnant women in Turkana County, at only 17.2% and their knowledge on key obstetric danger signs was equally low across all the obstetric phases, namely pregnancy (13.5%), labor and childbirth (8.9%), and post-partum (21.9%). Key factors influencing practices of birth preparedness and complication readiness (BPCR) were found to be knowledge of obstetric danger signs during pregnancy, occupation (being housewife), having received health education on how to prepare for birth and its complications during antenatal care clinics, having received health education on danger signs of pregnancy, childbirth and after birth at the antenatal care clinics as well as community assisting pregnant women in whichever way to prepare for birth and its complications. Lack of awareness of obstetric danger signs among the pregnant women and even among their partners, limited healthcare access due to insecurity along the roads and distant health facilities and hospitals as well as lack of sufficient health services, affordability of the available means of transport, low economic status, and cultural practices were identified through focus groups and key informant interviews as key factors limiting BPCR practices among pregnant women in the study area.

### What is known about this topic

- The birth preparedness and complication readiness practices among pregnant women are low;
- Awareness of danger signs among pregnant women is also low;
- Occupation (being a housewife), knowledge of danger signs during pregnancy, receiving health education on birth preparedness and danger signs at ANC, and being assisted by the community in whichever way are associated with being prepared for birth and its complications.

### What this study adds

- The findings showed that using diverse methods to communicate birth preparedness, complication readiness, and obstetric danger signs is essential to improve maternal health outcomes.

## Annex

**Annex 1**: summary of qualitative findings (PDF - 119KB)
